# Mental health literacy among Arab-Palestinians in Israel and its association with socioeconomic characteristics and healthcare utilization

**DOI:** 10.1186/s13584-026-00789-6

**Published:** 2026-10-29

**Authors:** Noor Abo-Ras, Rafael Youngmann, Efrat Neter

**Affiliations:** 1 Ruppin Academic Center, Clinical Psychology Graduate Program, Emek Hefer, Israel; 2 Department of Behavioral Sciences, Ruppin Academic Center, Emek Hefer, Israel

**Keywords:** Mental health literacy, Mental health service utilization, Palestinian citizens of Israel

## Abstract

**Background:**

Research on Mental Health Literacy (MHL) has expanded across cultural minority groups, yet little is known about the relationships between socioeconomic status (SES), MHL, and mental health (MH) service utilization. This study investigates the relationship between MHL, SES, and MH service utilization among Palestinian citizens of Israel (PCIs).

**Methods:**

A cross-sectional survey (*n* = 372, M_age_ 36.88 ± 13.52 years, 68% female), based on the Australian National Survey of Mental Health Literacy and Stigma, examined associations between SES indicators (education and income), MHL components, and MH service utilization. Compared to the PCI population, the sample included more women, older people, and more people with higher education.

**Results:**

Higher SES, as assessed by both indicators, was significantly associated with greater mental health literacy (MHL) at the bivariate level, particularly in terms of accurate disorder identification, knowledge of appropriate interventions, and lower levels of stigma. Hierarchical logistic regression revealed that education and income were significantly associated with MH service use (OR = 3.62, 95% CI: 1.38–9.45; OR = 0.36, 95% CI: 0.13–0.99, respectively), and first-aid MHL knowledge also contributed to explaining variance (OR = 0.86, 95% CI: 0.75–0.99). Other MHL indices, such as stigma and knowledge of potential helpers, were not significantly associated with service utilization.

**Conclusions:**

The findings underscore the interplay between structural inequalities and cultural beliefs in shaping MH knowledge and behavior, and highlight the need for culturally sensitive, socioeconomically accessible interventions to improve MH outcomes in minority populations. The authors advocate for integrating MHL education into secondary educational settings and implementing policy reforms that address both material disparities and cultural barriers to care. These insights contribute to the global discourse on MH disparities in indigenous and marginalized communities.

## Introduction

Mental health literacy (MHL) refers to the knowledge and attitudes that help recognize, manage, and prevent mental health (MH) issues [1, 2]. The concept has expanded to include understanding how to achieve and maintain positive MH, reduce stigma, and improve help-seeking behaviors [3]. The assessment of MHL encompasses the recognition of psychological distress, knowledge of treatment options, self-help strategies, MH first-aid skills for supporting others in distress, and stigma-related attitudes towards people with MH conditions [2].

### Social and cultural determinants of mental health and their relation to service utilization

Social and structural determinants, such as gender, socioeconomic status (SES), and racism play a crucial role in shaping MH outcomes and creating significant health disparities [4]. Research has demonstrated that systemic inequities, including limited access to quality healthcare and education, increase the risk of MH issues in marginalized communities [5]. Furthermore, prolonged exposure to discrimination-related stress is associated with higher levels of depression, anxiety, and substance use [6].

### Socio-cultural determinants of mental health

In many communities, MH conditions are stigmatized, and symptoms are often attributed to spiritual or moral failings rather than medical factors, leading to the avoidance of professional help [7, 8]. While strong social support networks and cultural practices can promote well-being [9, 10], studies have also demonstrated that interventions aimed at improving MHL and reducing MH-related stigma are associated with better help-seeking behaviors, greater service utilization, and improved MH outcomes, including among socioeconomically disadvantaged and resource-limited populations [10, 11]. A lack of culturally competent care often results in inaccurate diagnoses for minority populations [12], highlighting the need for policy reforms that address both systemic inequalities and deeply rooted cultural norms and values (such as concerns about family honor and tendencies toward somatization) to promote equitable outcomes [13, 14].

### The case of the Arab-Palestinian minority in Israel

Palestinian citizens of Israel (PCI) constitute 21.1% of the population. This minority population faces unique social-structural challenges. In 2021, over 50% of Arab families lived below the poverty line [15]. Studies have shown that SES is significantly associated with their health outcomes, with social participation serving as a mediator between SES and both self-rated health and MH in early to late midlife and older adults [16].

Beyond being a minority, PCIs are situated within an ongoing political conflict, which imposes substantial stress on their mental well-being [17, 18]. The PCI community is characterized by collective values, loyalty to family, and strong support systems; however, these cultural and religious beliefs often clash with the bio-psycho-medical model that shapes Israel’s healthcare servicesm [13, 19]. Thus, despite relatively high MHL, as reflected in mental disorder identification [20] and the potential for strong communal support, cultural and religious beliefs, as well as MH stigma, continue to impede the utilization of formal MH services [17, 20–22]. Mental health service utilization is further constrained by geographic and ethnic inequities, which exacerbate disparities in access to care and contribute to the treatment gap [7, 23].

### Mental health literacy, service utilization, and previous findings in the PCI community

Low MHL is a significant barrier to accessing evidence-based treatments and is linked to a “treatment gap” [24, 25]. Limited public knowledge regarding MH support hinders social networks from providing necessary first aid or guiding individuals toward professional help [26, 27].

In Israel, PCIs and certain immigrant groups tend to exhibit lower MHL and more negative attitudes toward MH services than veteran Jewish Israelis [17, 28, 29]. The inconsistencies observed in the literature on PCI (see below) likely reflect sociodemographic variations across sampled populations, as well as the multidimensional nature of MHL. Traditional populations often endorse somatic and culturally rooted explanations of illness rather than the Western biopsychosocial model and typically rely on informal familial support networks [17, 19]. In contrast, studies reporting higher levels of MHL among PCIs generally involve younger and/or more highly educated participants who have greater exposure to formal healthcare systems and mental health discourse [30, 20]. Crucially, while PCIs demonstrate relatively high identification of specific mental disorders [20], significant disparities in service utilization remain [21, 22], especially in cases of severe psychopathology [30]. This treatment gap is largely driven by stigma-related and attitudinal barriers, which have been identified as major obstacles to the use of professional MH services among PCIs [22]. Furthermore, evidence from Israel and Europe indicates that such negative attitudes toward mental illness are especially pronounced in the context of severe mental disorders, such as schizophrenia [31, 32].

A critical research gap exists regarding the specific association of socioeconomic indicators on MHL and mental health service utilization in this population, particularly in comparison to Jewish citizens [23]. Although prior research by Abo-Rass [30] and Abu-Kaf examined the link between MHL and service utilization among PCIs, income was not found to be a significant predictor of utilization in their final regression model. Furthermore, while SES variables have been examined previously in the context of mental health service utilization, they have not been investigated in relation to MHL components themselves [17, 30]. This study addresses these gaps by explicitly examining how major indicators of SES - education and income - relate to both MHL and mental health service utilization.

### Study aims and hypotheses

The current work focuses on MHL among PCIs and its association with healthcare utilization, with a specific emphasis on socioeconomic indicators. We hypothesize that:

#### H1

Social-structural variables (income and education) are significantly associated with various indices of MHL.

#### H2

Both MHL components and social-structural characteristics are associated with the utilization of MH services.

#### H3

MHL contributes significantly to the explained variance of MH service utilization, beyond the contribution of background and socioeconomic variables.

## Methods

### Participants and procedure

The study involved 372 participants, of whom 204 responded through an Arabic-language internet panel, and 168 responded to an online survey disseminated via social media (e.g., Facebook, WhatsApp) with no monetary compensation, as well as distributed in a printed mode through a “snowball” sampling method, the latter among older adults. In the online mode, participants were provided with a link to a Qualtrics survey and invited to complete it online. To ensure data quality among the non-panel participants, IP addresses were examined to identify duplicate responses or unusual response patterns and to verify that the data did not include automated submissions (e.g., by bots). Sample size adequacy was determined using the formula *N* > 50 + 8P, where P represents the number of predictors [33]. Given that the study included 12 predictors in total (eight background variables and four MHL indicators), the minimum required sample size was 146 participants.

Eligible participants were Arabic-speaking adults (aged 18 years or older) who completed at least 80% of the survey. Individuals who did not meet these eligibility criteria were excluded from the analyses.

Participants were notified that the survey was part of a psychological study on “Mental Health Literacy”, gave active consent to participate anonymously, and were guaranteed that no identifying information would be disclosed if the study was published.

The study was approved by the Institutional Review Board of Ruppin Academic Center (IRB) (approval 2022 –204 S/CP) and was registered (https://osf.io/tbry9/overview*).*

### Design

The study design was cross-sectional. The dependent variables included MHL and MH service utilization. The independent variables consisted of background variables such as age, gender, socioeconomic status (income adequacy and education), religion and religiosity, place of residence, and self-reported health. In one analysis where the dependent variable was MH service utilization, the independent variables included MHL, self-reported health, and background variables.

### Materials

***Background variables*** - Participants were asked to indicate their gender, age, self-reported health, perceived income adequacy [34], place of residence, religion (Christian, Muslim, Druze), religiosity, and education (originally including 8 response options, merged into four levels in some analyses). A subjective measure of income adequacy was selected because individuals’ perceptions of whether their financial resources are sufficient often explain unique variance in health and psychological outcomes beyond traditional indicators such as income, education, or occupation, making them especially useful in behavioral research [35]. Self-reported health was included as a key indicator of overall health status because it provides a comprehensive assessment that integrates physical, psychological, and functional aspects of health. Despite its simplicity, self-rated health has consistently been shown to predict morbidity and mortality independently of objective health measures and is therefore considered a robust indicator of population health and well-being [36].

***Mental health literacy*** - MHL was assessed using items from the Australian National Survey of Mental Health Literacy and Stigma [37]. **The Australian National Survey of Mental Health Literacy and Stigma (NSMHLS)**, conducted in 2003–2004, was a landmark population survey designed to assess Australians’ knowledge and beliefs about mental disorders, their views about appropriate treatments, help-seeking preferences, and stigma toward people experiencing mental illness. In the original survey, respondents were presented with short **vignettes** describing individuals meeting criteria for specific mental disorders (e.g., depression, schizophrenia) and were then asked a series of questions (see below). We chose this approach as it was less directive than other existing measures. In the current study, three MH conditions with varying prevalence were presented: depression and PTSD (both common prevalence), and psychosis/schizophrenia (low prevalence). Each participant randomly received two case descriptions, one low (schizophrenia) and one high prevalence (either depression or PTSD). Survey length was deliberately restricted to reduce participant burden, limit fatigue-related response bias, and enhance completion rates.

Subsequent to each vignette, participants were first asked to identify the MH condition presented in the vignette. They were then asked whether potential helpers (e.g., family member, friend, doctor, psychologist), first aid courses of action (9 types of help), and interventions (12 types) were helpful, harmful, neither helpful nor harmful, depending, or “don’t know”. Lastly, they were asked about stigma towards the person in the vignette.

Scores for each component of mental health literacy (MHL) were computed separately for each variable and MH condition, followed by the construction of composite indices. Identifying MH problems was coded as accurate or incorrect for each of the three conditions; as each person read two vignettes, the variable ranged from 0 to 2. Literacy variables for knowledge concerning potential helpers, appropriate interventions, and first aid were calculated as follows: initially, a licensed clinical psychologist, licensed clinical social worker, and three clinical psychology graduate students classified accurate responses for each condition and each variable (helpers, interventions, first aid). MHL was then calculated by summing the accurate responses for each variable, for each condition, and across the conditions. For example, in the case of schizophrenia, an accurate response for the potential helper category “psychiatrist” was considered “helpful.” Similarly, an accurate response for the potential helper “close friend” was classified as “neither helpful nor harmful” or “depends” (see details on items and scoring in Supplemental Material).

### Data analysis

First, study participants were described, and descriptive statistics were computed for MHL indices, the prevalence of seeking information about MH, and the prevalence of using MH services. MH service uptake (used vs. not used) was further described by participants’ background characteristics. Next, SES (income adequacy and education) differences and associations with MHL indices were assessed using either an independent-samples t-test, a chi-square test, or association measures (Pearson or Spearman), depending on the variable type. Finally, a two-step hierarchical logistic regression analysis was performed to assess the extent to which background characteristics and MHL were associated with mental health service utilization. In the first step, background variables - age, sex, education, and income adequacy - were entered; in the second step, MHL measures were added. All data analyses were performed using SPSS software.

## Results

### Participants’ characteristics

A total of 547 individuals clicked on the online questionnaire. Participants who answered less than 80% of the questions were excluded from the analysis. Among the participants (*n* = 372, see Table 1), 253 were women (68.0%), 108 were men (29.0%), and 3 identified as ‘other’ (0.8%). The mean age was 36.88 years (SD = 13.52), with ages ranging from 18 to 101. Most participants were Muslim (*n* = 302, 81.2%), and the majority resided in central Israel (*n* = 184, 49.5%). Most participants had higher education, and in terms of economic status 65.1% reported that they manage with their income or live comfortably, while approximately one-third (35.0%) indicated that they struggle or struggle significantly to make ends meet. The majority of participants reported being in good health. Over half of the participants described themselves as religious or very religious (59.2%), while about 40.9% identified as non-religious or slightly religious. Less than half the sample (42.5%) sought information on MH in the past year, and slightly more than a fifth used MH services (22.3%).

The third column of Table 1 presents the demographic data of a nationally representative sample from the Central Bureau of Statistics (CBS) for the Arab-Palestinian population in Israel. As can be seen, our sample had more women, was older, and was more educated than the representative national sample.

The table also reports on the above characteristics by mental health service utilization (Yes/No). Both the socio-structural indicators of education and income adequacy were significantly associated with MH service use (the more educated, the more utilization, and the more comfortable with income, the less utilization), as well as the degree of religiosity (the more religious, the less service use) and self-rated health (the healthier, the more MH service use).

**Table 1:** Participants’ characteristics and mental health services utilization

Characteristics	*N* (%) or Mean (SD)	CBS^a^ data on the general Arab Palestinian population (%)	MH-SU: Yes *N* (%) 83 (22.3)	MH-SU: No *N* (%) 289 (77.8)	Statistic / *p*-value
**Gender**					*χ² = 0.93; p = *.33
Women	253 (68.0)	50.9	(24.1)	(75.9)	
Men	108 (29.0)	49.7	(19.4)	(80.6)	
Other	3 (0.8)				
Missing	8 (2.2)				
**Age, Mean**	(*M* = 36.88, SD = 13.52)	26.24	(M = 37.11, SD = 12.5)	(M = 36.82, SD = 13.72)	*t* = -017 *p* = .864
**Religion**					χ² = 0.58; *p* = .750
Muslim	302 (81.2)	85.2	(21.5)	(78.5)	
Christian	35 (9.4)	7.1	(25.7)	(74.3)	
Druze	35 (9.4)	7.5	(25.7)	(74.2)	
**Religiosity**			(M = 2.66, SD = 0.77)	(M = 2.44, SD = 0.84)	*t* = -2.28; *p* = .024
Very religious	26 (7.0)	-			
Religious	194 (52.2)	-			
Slightly religious	96 (25.8)				
Non-religious	56 (15.1)	-			
**Education** ^**b**^			(M = 2.94, SD = 0.93)	(M = 2.65, SD = 0.98)	*t* = -2.05; *p* = .03
Primary and secondary	63 (16.9)	37.7			
Post-secondary	58 (15.6)	22.1			
BA degree/ student	175 (47.0)	18.3			
MA degree and higher	23 (6.2)	14.6			
**Income adequacy** ^**b**^			(M = 2.59, SD = 0.94)	(M = 2.81, SD = 0.85)	*t* = 1.92, *p* = .029
Finding it very difficult	33 (8.9)	-			
Finding it difficult	97 (26.1)	-			
Managing	168 (45.2)	-			
Living comfortably	74 (19.9)	-			
**Self-rated health**			(M = 3.94, SD = 1.37)	(M = 4.3, SD = 1.25)	*t* = 2.32; *p* = .022
Very bad	3 (0.8)	-			
Bad	18 (4.8)	-			
Fair	89 (23.9)				
Good	150 (40.3)	-			
Very good	112 (30.1)	-			
**Searched for Mental Health Information**					χ² = 0.45.41; *p* < .001
Yes	158 (42.5)	-	(39.2)	(60.8)	
No	214 (57.5)	-	(9.8)	(90.2)	

Note. CBS = Central Bureau of Statistics; MH-SU = mental health service utilization

^a^ (Central Bureau of Statistics on Arab Population, 2020)

^b^ Significance testing on education and income were one-sided

### Bivariate associations between MHL indices and SES indicators (income adequacy and education)

Table 2 displays the associations between MHL indices and SES indicators. The association between the SES indicators themselves was *r*_s_(372) =  0.20, *p*<.001.

A significant weak positive Spearman association was found between accurate illness identification and both education andincome adequacy: *r*_s_(372) = 0.21, *p*<.001, and *r*_s_(372) = 0.11, *p* = .024, respectively. Similarly, the index of appropriate interventions was also associated with both SES indicators, *r* = .198, *p*<.001, and *r* = .106, *p* = .041, respectively. The general MH literacy index (across conditions and types of knowledge) was significantly associated with income adequacy *r* = .15, *p* = .005, so higher income was associated with higher literacy, but the overall index was not significantly associated with education. Lastly, negative stigma toward persons described in the vignettes was weakly associated with education, *r* = .19, *p*<.001, but unrelated to income adequacy. Knowledge about potential helpers and on first aid was unrelated to SES indicators.

Ad hoc analyses examining the associations between other demographic characteristics and MHL indices revealed a significant negative correlation between age and accurate illness identification: *r*_s_(372) = − 0.169, *p* = .002. Additionally, differences in accurate illness identification were observed based on religion: *χ* ^2^ (4,372) = 18.396, *p* = .001, Cramer’s *V* = 0.222, so that the percentage of Christians who correctly identified the illness vignettes was the highest (62.9%), compared to Muslims (47.7%) and Druze (22.9%). Similarly, a significant positive correlation was found between stigma and degree of religiosity, with higher levels of religiosity associated with higher stigma *r* (372) = 0.14, *p* = .004.

**Table 2:** Correlation between MHL indices and SES indicators (income adequacy and education)

1. Illness identification ^a^	Income adequacy	Education
	0.11*	0.21**
2. Potential helpers	0.06	0.02
3. Appropriate interventions	0.20**	0.11*
4. First aid	0.10	0.10
5. General MHL knowledge index	0.14*	0.08
6. Stigma	0.00	0.19**

Note. **p < .01; *p < .05

^a^ Spearman correlation in this variable

### Utilization of mental health services: a multivariate analysis

To examine the variables associated with the utilization of MH services (0 = No, 1 = Yes) in relation to background characteristics and MHL, we conducted a hierarchical logistic regression analysis (Table 3). In the first step, demographic and background variables were entered: gender, age, education, and perceived income adequacy. In the second step, MHL variables were included: identification of MH conditions, knowledge of potential helpers, first aid, and appropriate interventions, and stigma (all were across conditions). In the first step of the demographic variables, the lowest category (primary and secondary education) was selected as the reference group. Income was also divided into three dummy variables, with the group “struggling significantly to live on the current income” chosen as the reference group.

The first regression model was statistically significant *χ* ^2^ (8, *n* = 315) = 18.101, *p* = .020, with 8.2% of the variance in the utilization of MH services explained by the predictors (Nagelkerke R^2^ = 0.082). As shown in Table 3, the variables significantly associated with the utilization of MH services were socioeconomic status indicators, namely educational attainment and perceived income adequacy. Individuals with perceived higher income (“managing with income”) were less likely to use MH services than those who described themselves as “struggling significantly to live on their current income”, OR = 0.36, 95% CI = [0.15,0.89]; and likewise, “living comfortably on my current income” OR = 0.36, 95% CI = [0.13,0.99]. Conversely, individuals with higher educational attainment (a master’s degree or above) were more likely to use MH services than those with primary education or partial completion of secondary education, OR = 3.62, 95% CI = [1.38,9.45]. The variables of age and gender were not found to be associated with the utilization of MH services.

The second model was also statistically significant, *χ* ^2^ (13, *n* = 318) = 23.789, *p* = .033, with 10.7% of the variance in the dependent variable explained by the predictors (Nagelkerke R^2^ = 0.107). Knowledge of first aid was significantly associated with the utilization of MH services, OR = 0.864, 95% CI = [0.75,0.99], so that greater knowledge of first aid interventions was associated with lower utilization of MH services. The SES indicators remained significantly associated with MH utilization in this step. Education remained significant, with individuals with higher education (master’s degree or above) being more likely to use MH services compared to those with ‘primary or partial/complete secondary education’ OR = 3.75, 95% CI= [1.38,10.16]. Perceived income also remained significantly associated with MH use, with individuals who described themselves as ‘living comfortably on my current income’ being less likely to use MH services OR = 0.35, 95% CI = [0.14,0.87], as well as those ‘living comfortably on my current income’ OR = 0.35, 95% CI = [0.13,0.99], compared to those who described themselves as ‘struggling significantly to live on their current income’. The variables of age, gender, recognition, knowledge of potential helpers, appropriate interventions, and stigma were not found to be significantly associated with the utilization of MH services in the second model.

**Table 3:** Hierarchical logistic regression of mental healthcare utilization explained by background and MH literacy variables

Variable	Model 1		Model 2	
	Odds ratio with 95% CI	*P*-value	Odds ratio with 95% CI	*P*-value
Gender	1.41 [0.73, 2.71]	0.301	1.46 [0.75, 2.84]	0.263
Age	1.01[0.98,103]	0.665	1.01 [0.98, 1.03]	0.502
Education				
Primary and secondary (reference)				
Post-secondary	2.04 [0.75, 5.52]	0.163	1.93 [0.70, 5.34]	0.205
BA student/ degree	1.53 [0.62, 3.78]	0.354	1.49 [0.59, 3.75]	0.393
MA degree and higher	**3.62 [1.38**,** 9.45]**	0.009*	**3.75 [1.38**,** 10.17]**	0.009*
Income				
Very difficult (reference)				
Difficult	0.78 [0.31, 1.96]	0.601	0.77 [0.34, 1.94]	0.578
Managing	**0.36 [0.15**,** 0.89]**	0.026*	**0.35 [0.14**,** 0.87]**	0.024*
Living comfortably	**0.36 [0.13**,** 0.99]**	0.047*	**0.35 [0.13**,** 0.99]**	0.049*
Knowledge of potential helpers			1.00 [0.95, 1.05]	0.956
Appropriate interventions			1.030 [0.92, 1.14]	0.630
First aid			**0.864 [0.75**,** 0.99]**	0.042*
General stigma			1.030 [0.92, 1.14]	0.254
Recognition			0.90 [0.60, 1.36]	0.623
R^2^	0.082		0.107*	
R^2^ change			0.035	

Note. *P < .05; **P = .01

## Discussion

The present study examined mental health literacy (MHL) among PCIs, with a specific focus on the relationship between social-structural variables such as education and socioeconomic status (SES). Prior research has consistently shown that individuals with higher SES tend to possess greater MH knowledge and are more adept at recognizing mental conditions [2, 38]. Education, in particular, has been identified as a central predictor of MHL, potentially through its contribution to the development of general knowledge and the facilitation of access to MH-related information.

In line with these findings, the current study revealed a weak but statistically significant association between SES and the accurate identification of MH conditions (H1). It also found inconsistent associations with other knowledge indices and stigma, and a consistent lack of association between SES and knowledge of potential helpers. In summary, H1 was partially supported. Regarding mental health service utilization, the study showed that SES and MHL together accounted for 10.7% of the variance in service use (supporting H2), and that MHL added to the explained variance in service utilization (thus supporting H3), underscoring the importance of these variables in shaping MH-related behaviors. Still, most of the variance in MH service utilization remained unexplained in the current study.

The present findings on SES indicators echo those of Steele et al. [39], who reported that in Canada, individuals with higher education were substantially more likely to access a range of MH services, including psychiatrists (15%), general practitioners (12%), psychologists (16%), and social workers (16%), than those with lower educational attainment.

Our findings align with and expand upon prior research in the PCI population. A similar study conducted by Abo-Rass and Abu-Kaf [30], which used a different, more structured measure of MHL, also identified a significant relationship between MHL and service utilization, with a comparable level of explained variance to the background variables, which included gender and education (7% vs. 10% in the present study). However, Abo-Rass and Abu-Kaf [30] reported a higher proportion of explained variance, mainly driven by the inclusion of psychological distress, which contributed an additional 23% to Cox and Snell’s R². The MHL indices in that study contributed a further 8%, yielding a total explained variance of 38%. Finally, different indices of MHL were associated with MH service utilization in the current study and in Abo-Rass and Abu-Kaf [30]. In the multivariate analysis of the current study, only the index of knowledge on MH first aid was significantly associated with MH service utilization. This finding may reflect proactive knowledge acquisition and greater contextual familiarity with psychological distress among highly educated minorities living under chronic stress. Such resources may enable individuals to engage in informal coping strategies and self-regulation before seeking formal clinical care [28]. In contrast, three indices of MHL found to be significantly associated in the Abo-Rass and Abu-Kaf’s study [30], specifically knowledge of professional help, knowledge of where to seek information, and attitudes promoting recognition or help-seeking behaviors. It is possible that the measure used in the latter study was more sensitive and/or valid.

The lack of an association between stigma and mental health service utilization was unexpected. This finding contrasts with results reported in two Arabic-speaking samples [13, 40], but is consistent with findings from another Arabic-speaking sample [22]. Our results may be interpreted in light of the sample’s high educational attainment, as participants may harbor less stigma than individuals in more representative population samples. In addition, it is worth distinguishing between different kinds of attitudes, specifically stigma towards people with MH conditions, on the one hand, and attitudes promoting recognition or help-seeking behaviors, on the other hand.

Importantly, while both income and education are core components of SES, they may shape access to MH care in different ways. Higher educational levels may reduce stigma, enhance MH awareness, and normalize help-seeking behaviors, particularly when acquired through Westernized Israeli academic institutions, where the discourse of MH and resilience is prevalent. Such exposure may facilitate a shift in cultural norms within the Palestinian-Arab community, making MH care more acceptable. In contrast, though higher income may reduce structural barriers such as cost and availability, enabling individuals to allocate time and resources toward seeking therapy and investing in their well-being [41], it may also reduce service uptake due to reduced exposure to stress and thus reduced psychological distress.

Despite these insights, much of the existing research on minority MH lacks a specific focus on indigenous minorities, making the current study’s contribution particularly significant. By focusing on the Palestinian-Arab minority in Israel - a group with unique cultural, linguistic, and political characteristics - this research adds depth to the existing empirical literature on MHL and service use among Arab populations in Israel. It also demonstrates the necessity of understanding the nuanced intersections of culture, SES, and MH.

### Strengths and limitations

The present study has notable strengths. First, its focused approach on the Palestinian-Arab minority in Israel yielded valuable insights into the interplay between SES and MHL regarding service utilization within a distinct sociopolitical context. Second, the study utilized objective and validated instruments to evaluate MHL, thus extending the analysis beyond subjective perceptions of literacy. Nevertheless, the study is not without limitations. First, the study did not assess psychological distress, which was found to moderate MH service use [21]. Second, the use of a convenience sample in the current study resulted in a skewed sample in terms of age, gender (more women), income and education, which limits the generalizability of the findings. Third, future studies are advised to utilize the recently adapted and validated tool of IMHL [20]. Addressing these limitations could potentially replicate and fortify the present findings. Lastly, the cross-sectional design of the study precludes inferring about causal relationships between SES, MHL and mental health service utilization.

### Conclusions and policy recommendations

This study investigated the complex interplay of MHL and SES, specifically education and income, in explaining mental health service utilization among PCIs. Our findings confirm that SES and MHL are both independently and jointly associated with the uptake of professional care, together accounting for 10% of the variance in service use. Specifically, higher education appears crucial, potentially by facilitating exposure to Westernized MH discourse, which may mitigate cultural stigma and promote the normalization of help-seeking.

The experience of the PCI community, an ethnic and religious minority facing socioeconomic disadvantage and chronic political stress, serves as a critical case study for understanding global MH disparities. Consistent with research on other marginalized groups, our findings highlight the persistent effects of cultural stigma and structural barriers. Consequently, a comprehensive, multilevel intervention strategy is essential, requiring a dual focus on both MHL promotion and structural equity.

A key recommendation emerging from this work is the integration of evidence-based MH education, such as that grounded in cognitive-behavioral therapy with a preventive approach [42], into high school curricula, particularly in communities with lower educational attainment, and into culturally sensitive digital MH platforms. Introducing MHL concepts early could raise awareness, reduce stigma, and improve access to resources, equipping individuals with the knowledge and tools necessary to navigate MH challenges [43]. Digital tools can be culturally adapted and offer high accessibility at low cost, thus helping to mitigate the structural and cultural barriers to accessing formal services identified in this research. Such efforts could help foster more informed and supportive environments and improve overall community wellbeing.

MH policymakers in the Ministry of Health, in cooperation with the Ministry of Education, should consider dedicated and equitable resource allocation to support MHL integration across all PCI high schools, with special attention to communities characterized by lower SES. Furthermore, policy development should prioritize the support and promotion of culturally and linguistically adapted digital MH platforms as a low-cost means to address structural and geographical access barriers.

## Data Availability

The datasets generated and/or analyzed during the current study are available in the OSF repository ( [https://osf.io/tbry9/overview](https:/osf.io/tbry9/overview) ).
